# Polar Expansion Dynamics in the Plant Kingdom: A Diverse and Multifunctional Journey on the Path to Pollen Tubes

**DOI:** 10.3390/plants2010148

**Published:** 2013-03-18

**Authors:** David S. Domozych, Chelsea Fujimoto, Therese LaRue

**Affiliations:** Department of Biology and Skidmore Microscopy Imaging Center, Skidmore College, Saratoga Springs, New York, NY 12866, USA; E-Mails: cfujimot@skidmore.edu (C.F.); tlarue@skidmore.edu (T.L.)

**Keywords:** polar expansion, plants, cell wall, actin, Golgi, pectins

## Abstract

Polar expansion is a widespread phenomenon in plants spanning all taxonomic groups from the Charophycean Green Algae to pollen tubes in Angiosperms and Gymnosperms. Current data strongly suggests that many common features are shared amongst cells displaying polar growth mechanics including changes to the structural features of localized regions of the cell wall, mobilization of targeted secretion mechanisms, employment of the actin cytoskeleton for directing secretion and in many cases, endocytosis and coordinated interaction of multiple signal transduction mechanisms prompted by external biotic and abiotic cues. The products of polar expansion perform diverse functions including delivery of male gametes to the egg, absorption, anchorage, adhesion and photo-absorption efficacy. A comparative analysis of polar expansion dynamics is provided with special emphasis on those found in early divergent plants.

## 1. Introduction

**Figure 1:**
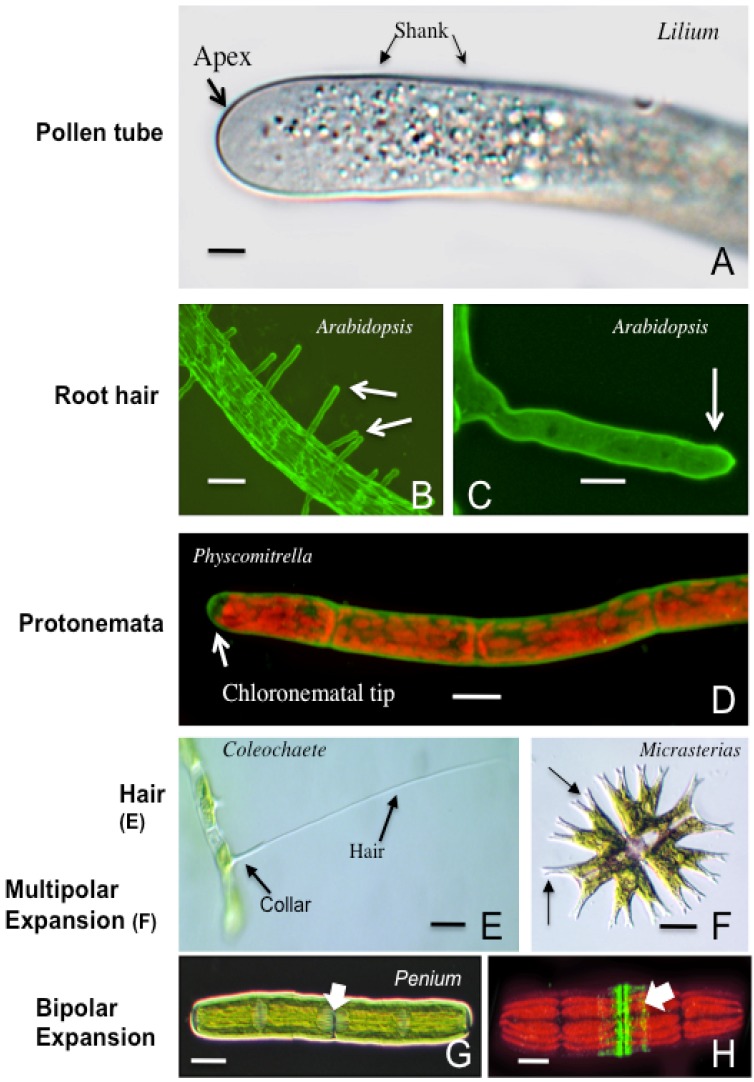
The diverse range of polar expansion mechanics in plants. (**A**) highlights the vesicle-rich zone at the apex of a *Lilium* pollen tube. The shank constitutes most of the pollen tube. Differential Interference Contrast (DIC) microscopy image. Bar = 6 µm; (**B**) shows the emergence of root hairs (arrows) from the root of *Arabidopsis* labeled with the anti-xyloglucan antibody, LM15. Confocal Laser Scanning Microscope image (CLSM). Bar = 100 µm; (**C**) is a magnified view of the root hair labeled with LM15. CLSM image. Bar = 20 µm; (**D**) displays the chloronemata stage of the gametophyte phase of the life cycle of *Physcomitrella* labeled with the anti-HG antibody, JIM5. CLSM image. Bar = 10 µm; (**E**) is a hair cell or seta of *Coleochaete nitellarum*. Note the elongate hair as it emerges from the basal collar. DIC image. Bar = 25 µm; (**F**) displays the multi-lobed nature (arrows) of the desmid, *Micrasterias*. These lobes are products of multi-polar expansion. DIC image. Bar = 10 µm; (**G**) shows the two semicells surrounding a central isthmus (arrow), the site of pre-division expansion in *Penium*. DIC image. Bar = 8 µm; (**H**) is a JIM7-labeled (sp. = high esterified HG) *Penium* in a pre-division expansion. Note the two bands at the isthmus (arrow), the site of the bipolar growth mechanism. CLSM image. Bar = 5 µm.

The developmental dynamics of pollen tube growth represent a spectacular example of anisotropic cell expansion in eukaryotes. This growth mechanism entails finely-tuned and highly-coordinated interactions of the tube’s cell wall biosynthetic and secretory machinery with the cytoskeletal system as well as multiple, cross-talking signal transduction pathways [1]. These activities are focused at a precisely defined “softened” (*i.e.*, loosened) zone in the cell wall located at the pollen tube tip [2]. Expansion is ultimately driven by non-vectorial turgor pressure on this softened zone and manifests in a tip-growing (*i.e.*, polar expanding) cylinder. The growth of the pollen tube is essential for reproduction as it creates a path for sperm cells or nuclei on their journey to the female gametangium for subsequent fertilization of the egg. Polar expansion though, is not unique to pollen tubes. In vascular plants, root hairs develop by a comparable polar expansion mechanism that leads to structures that are used for water and mineral absorption, anchorage, and communication with soil microbiota. In ferns, “rhizoids” also grow via polar growth and serve as anchors and absorption conduits. Polar expansion though is even a more widespread phenomenon in early divergent (*i.e.*, “ primitive”) plant taxa as is exemplified by the protonemata of mosses, the rhizoids and protonemata of the Charales (Charophycean Green Algae, CGA, or Streptophyta), the setae of the Coleochaetales (CGA) and the symmetrical growth patterns exhibited by desmids (Zygnematales) (Figure 1). While many of the subcellular processes that yield these polar expansion products in plants are similar, a range of unique subcellular mechanisms, specific to the taxon/cell type and directly responding to particular environmental triggers, has evolved in these early divergent plants. In this paper, a comparative analysis of the mechanisms involved in polar expansion phenomena is presented. 

## 2. Common Components and Processes Associated with Polar Expansion in Plants

Though the environmental signals to which polar-growing cells respond may differ greatly (e.g., gravity, light, nutrient gradients, chemical gradients in reproductive tissue) and the substrates through which they grow may vary significantly, many of the underlying molecular, biochemical and subcellular mechanisms that are activated to yield polar expansion appear to be similar throughout green plants including early divergent taxa. These include:

### 2.1. Cell Wall

The cell wall and its inclusive composite of polymers represent key structural components involved in polar expansion. Precise geographic and temporal deposition of wall polymers or a remodeling of wall polymers in the wall at a specific locus of the cell is a major mechanism in directing polar growth. This, in turn, requires a significant investment of the organism’s genetic machinery. For example, in *Arabidopsis*, approximately 1/3 of the 800 genes related to cell wall synthesis are found in pollen with the functions of most unknown [2,3]. Those specific wall components that have been recognized as major parts of the process include: 

#### 2.1.1. Pectin and Pectin Methylesterase (PME)

Pectin represents a class of galacturonic acid (GalA)-containing matrix polymers that are integrral to the structural integrity of the cell wall [4,5,6]. There are several types or classes of pectin including the homopolymeric, homogalacturonans (HGs) and the heteropolymeric rhamnogalacturonans, RG-I and RG-II. HGs represent the most common and well-studied pectin type in polar expansion phenomena, most notably in pollen tubes [2,7]. HGs are synthesized in the Golgi body, packaged and transported in exocytic vesicles and secreted to the apoplast at the expanding growth tip. The secreted HG is often in a high methyl-esterified form that is strong enough to resist turgor pressure at the growth tip but is flexible so as to allow for regulated expansion. After secretion, the high methyl-esterified HG is de-esterified by pectin methyl esterase (PME) that in turn, exposes the negatively-charged carboxyl group on the C6 of the GalA residues. Ca^2+^ may then crosslink the GalAs of adjacent HG chains that results in the formation of a rigid gel. This process occurs subapically at the shoulder (*i.e*., the beginning of the shank of the pollen tube). This pectin-Ca^2+^ dynamic is also central to the expansion mechanisms of other polar growing cells including desmids of the CGA (see Section 3). However, pectin remodeling and positioning in the wall microarchitecture may affect wall and cell expansion in additional ways. For example, the de-methyl-esterification of HG also releases H^+^ ions that may subsequently cause acidification of localized wall zones that then activates other wall-modifying agents. Also, pectin may affect the structural dynamics of the cell wall by mechanically altering the movement of other wall polymers (e.g., hemicelluloses, proteoglycans; [5]). Likewise, pectin porosity and/or gelling changes after secretion may affect water movement [8] and in turn, the movement of expansion-associated expansin, XTH or other wall-modifying agents. In addition to HG, RG-I containing long (1 to >5) arabinan side chains may also affect expansion by regulating HG packing and polymer-polymer interactions in the cell wall [9].

PME is the enzyme that is integral to the pectin-Ca^2+^ mechanism in many polar expanding cells and is also involved in other phenomena such as cellular adhesion, stem elongation, fruit ripening, pollen and seed germination and root development [10,11]. PME is located at the polar tip of pollen tubes and is responsible for modifying (*i.e*., de-esterifing) recently-secreted high methyl-esterified HGs that, in turn, allow for cation (e.g., Ca^2+^)-crosslinking and accompanying rigidification of the cell wall. PME is believed to be regulated intramolecularly via its pro-region that inhibits its catalytic activity. This may prevent secreted pectins from being prematurely de-esterified and/or correctly targeting PME to its site of secretion in the cell wall. PME is also regulated by the PME inhibitor (PMEi), which shares some homology with the pro-region of PME and acts in a pH-dependent manner by partially obscuring the active pectin-binding site of the enzyme [12,13]. PMEis are thought to be active at the tip of pollen tubes where they prevent PME catalytic activity and this in turn, allows the tip to remain less rigid with high methyl-esterified HG. In the shoulder of the tube tip, PMEi is endocytosed, leaving PME to de-esterify the HGs and allowing Ca^2+^-crosslinking-based rigidification of the HG to occur [6,7]. PME activity releases protons and methanol that may also trigger a negative feedback mechanism [13].

#### 2.1.2. Cellulose, Callose and Xyloglucans

In addition to pectins, other wall polymers play critical roles in polar expansion dynamics. Cellulose microfibrils represent the main load-bearing component of most plant cell walls and function in polar growth phenomena. Often found in low amounts in pollen tubes (e.g., 10%), cellulose microfibril orientation likely controls the direction but not the degree of polar expansion [14,15]. It may also be that cellulose contributes to limiting the diameter of the pollen tube that results in a lower surface area that enhances growth through the female tissue. Additionally, cellulose may act as a stabilizing agent in the tube tip as it interacts with other polymers such as xyloglucans [9]. The cellulose synthesizing enzyme, cellulose synthase or CesA, is processed in the Golgi Apparatus and is transported in vesicles to the tube apex via the actin-mediated cytoplasmic streaming mechanism [16]. In root hairs of *Arabidopsis*, randomly oriented fibrils of (1-4)-ß-glucan, and not the typical crystalline microfibrils, are found at the growing tip. The protein, CLSD3, a (1-4)-ß-glucan synthase found at the plasma membrane of the expanding tip may be responsible for production of these randomly oriented fibrils [16]. The functional role of these polyglucans in root hair tip growth has not yet been resolved. Localized regions’ cellulose synthesis also plays a role in the polar morphogenesis observed in desmids and in bryophyte protonemata (Section 3).

Callose or ß-(1-3)-glucan, is also a major glucan found in polar expanding cells like the pollen tube where it forms the basis of the inner layer of the shank cell wall as well as the periodic cross walls that restrict backward movement of the sperm cells/nuclei. Callose synthase (CalS) is also processed via the Golgi-vesicle-actin cytoskeleton network [17]. Callose is also present in the cell walls of wound-stimulated rhizoids in the CGA, *Spirogyra*.

Xyloglucans are hemicellulosic polymers that form tight, non-covalent, H-bonds with cellulose microfibrils. This property allows for a tethering of the microfibrils and makes xyloglucans important regulators of microfibril slippage during wall expansion. Xyloglucan modulations leading to this wall “loosening” are often catalyzed by enzymes like xyloglucan endotransglucosylase or XET [18,19,20]. In root hairs, XET activity is always associated with root hair initiation suggesting an important role of xyloglucans in the polar growth mechanism. Xyloglucans, including highly acetylated forms, have been identified in pollen tube walls but their exact functional roles are not yet known [9].

#### 2.1.3. Arabinogalactan Proteins (AGPs)

**Figure 2:**
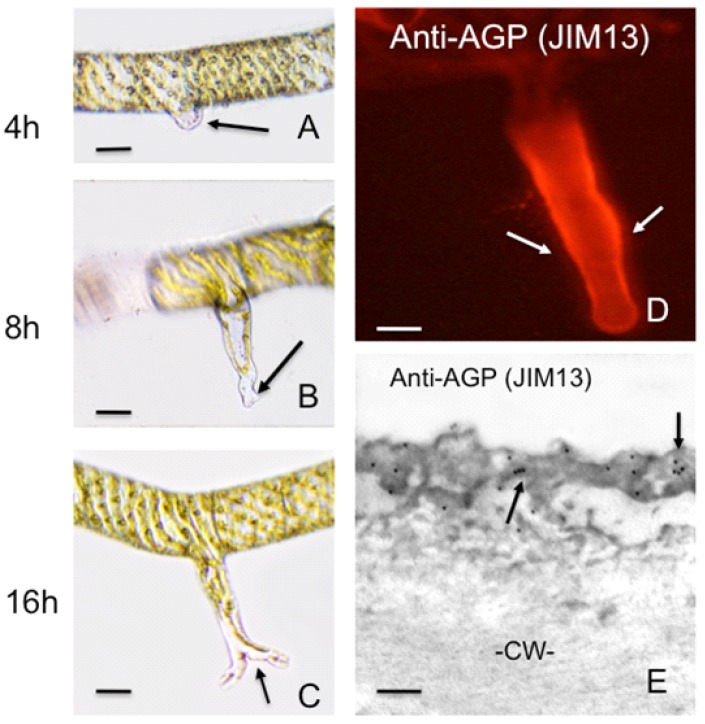
Wound-induced rhizoid formation in *Spirogyra*. (**A**) shows the emergence of a rhizoid (arrow) from the cell adjacent to a cell artificially wounded (*i.e.*, cut) 4 h earlier. DIC image. Bar = 20 µm; (**B**) displays the rhizoid 8 h after wounding. Note that branching occurs at the tip (arrow). DIC image. Bar = 20 µm; (**C**) shows the rhizoid after 16h and the extensive branching that has taken place. DIC image. Bar = 20 µm; (**D**) shows a rhizoid labeled with the anti-AGP antibody, JIM13. Note that only the rhizoid wall labels (arrow). CLSM image. Bar = 15 µm; (**E**) is a JIM13 immunogold labeling of the rhizoid wall, Note that a sheath (arrows) external to the main cell wall (CW). Transmission electron microscopy (TEM) image. Bar = 100 nm.

Arabinogalactan proteins (AGPs) constitute a non-enzymatic family of cell surface hydroxyproline-rich glycoproteins whose protein core is extensively *O*-glycoslylated [21]. The carbohydrate moiety of AGPs is rich in arabinose and galactose and may account for 90% of mass of these macromolecules. AGPs are found in the cell wall and apoplast and may be attached to specific microdomains of the plasma membrane via GPI anchors. AGPs appear to be common constituents of polar growing cells including the apex of pollen tubes [9] root hairs [22] and the apical cells of the protonemata of the moss *Physcomitrella* [23]. The proposed functional roles of AGPs in polar growth are indeed diverse. They may bind to pectins and subsequently modulate wall integrity and function during wall deposition and expansion. They may also act as co-receptors at the plasma membrane of the apical tip that sense extracellular signals and interact with transmembrane proteins such as ion channels or receptor kinases. Also, they may form an interactive network with cytoskeletal agents involved in the expansion process [21,24,25]. AGPs have been found associated with pore complexes on the outer cell wall of desmids [26] or associated with the cell walls of wound-induced rhizoids in *Spirogyra* (Figure 2). These observations suggest a major role in adhesion.

#### 2.1.4. Expansins

Expansins are a group of cell wall proteins that induce wall extension and stress relaxation [27,28,29] during plant cell expansion. Expansin activity disrupts the bonding of cell wall glycans (e.g., xyloglucans) to cellulose microfibril surfaces and to each other. This subsequently results in the displacement/slippage of the wall polymers during turgor-driven expansion. Expansins have been identified in green algae, bryophytes and vascular plants and comprise a large multigene family containing two specific subgroups, α-expansins (EXP) and β-expansins (EXPB) [30]. Both exhibit cell-wall loosening activity, however, α-expansins are hypothesized to control cell wall enlargement dynamics and acid-growth while β-expansins are most likely involved in wall-loosening associated with pollen tube penetration [29]. Expansins have also been identified in root hairs and root hair initiation sites [27,30].

### 2.2. Exo- and Endocytosis: Actin and Actin-Associated Proteins

Targeted exocytosis of cell wall components and new membrane is an integral feature of polar expanding cells. Ketelaar *et al.* [31] estimated that over 9,000 exocytic vesicles were utilized per minute in a growing *Arabidopsis* root hair and over 2,500 vesicles were utilized in its pollen tube. These vesicles deliver not only new membrane and wall polymers but also critical wall-synthetic or modifying enzymes. Additionally, this study also showed that 86.7% of the membrane of the root hair and 79.0% of the membrane of the pollen tube are recycled by endocytosis. Similar endomembrane system-based phenomena have been described or at least implicated in other polar growing cells and excellent reviews of the specifics are available [32,33,34,35,36,37,38,39]. Equally importantly, the motive force and directional transport of these vesicles are functions of the cytoskeletal system that includes actin, actin-associated proteins and microtubules [40].

#### 2.2.1. Actin

An extensive and dynamic network of actin microfilaments is responsible for the transport of various exocytic and endocytic components that constitute the complex membrane trafficking involved in polar expansion phenomena [41,42,43,44,45]. In angiosperm pollen tubes, long axially-aligned actin bundles in the tube shank transport vesicles to the subapical cytoplasm and back to the rear in a “reverse fountain” cytoplasmic streaming mechanism. The subapical zone consists of a network of shorter and thinner actin cables that constitute the “fringe” [46]. This region is the zone where vesicles collect and most likely is key in the regulation of expansion. A finer meshwork of microfilaments is found at the tip terminus. The total actin network at the apex of the tube is responsible for spatio-temporal coordination of vesicle targeting and closely interacts with tip-based ROP GTPase [47,48]. Similar actin-based networks and in many cases, highly elaborate cytoplasmic streaming mechanisms, are also major components of polar growth of other cell types including root hairs, moss protonemata, CGA rhizoids and desmid lobe formation [49,50,51,52,53,54].

#### 2.2.2. Actin Binding Proteins

Actin dynamics in polar expansion also entails a diverse set of actin-binding proteins. Many of these proteins have been found in polar expanding cells including pollen tubes, root hairs, protonemata, and rhizoids. Myosin, the protein required for movement of membrane-bound cellular constituents on actin cables, has been shown to be integral to vesicle movement in polar growth [41]. Formins are actin-nucleating proteins that accelerate the critical rate-limiting step of dimer and trimer formation from actin monomers during rapid actin polymerization. Membrane-anchored formin at the polar tip of pollen tubes stimulates actin assembly necessary for expansion [46]. Formin also plays a major role in root hair initiation and expansion [49]. Overexpression of formins in both systems significantly alters the polar expansion mechanism [55]. Profilins are low molecular weight proteins that bind to G-actin in 1:1 ratio and control the amount of polymeric actin. They may also bind to proteins with contiguous stretches of proline, like formin, and contribute to actin filament assembly [56]. Profilin-based activity has been clearly demonstrated in the polar expansion mechanisms associated with pollen tubes and root hairs [56,57]. Villin is a Ca^2+^-responsive protein that controls actin turnover and has also been described in both pollen tubes and root hairs [45,58,59]. Adenylate cyclase-associated protein or CAP1, binds to G-actin and also regulates levels of polymeric actin in pollen tube expansion [56]. Other actin binding proteins include the depolymerizing factor or ADF, a pH-dependent actin severing agent that may help cycle actin and, capping proteins which attach to plus ends of F-actin and prevents subunit loss and addition to these ends [49,57]. Actin related proteins (ARPs) such as those of the ARP2/3 complex are involved in the translation of directional prompts into polar expansion in protonemata [60] and bulge initiation in root hairs [61].

### 2.3. Microtubules

Though much less is known about their function, microtubules also contribute to some polar expansion phenomena [62,63]. Their roles may include the organization of CesA complexes into organized clusters that yield specific cellulose microfibril orientations necessary for polar growth and/or coordination between cellulose synthesis and delivery of proteins and other molecules in polar growth zones [1]. They also play a role in gravitropism-based polar growth in some CGA (Section 3).

### 2.4. Phosphoinositides

Phosphoinositides (PIs) represent a class of signalling lipids that are integral to the functioning of many physiological processes in eukaryotes [64,65] including polar expansion phenomena exhibited in plants. PIs are derived from phosphatidylinositol (PtdIns) whose inositol ring can be phosphorylated by lipid kinases at the D-3, D-4 and D-5 positions to yield PtdIns3P (PtdIns-3-phosphate), PtdIns4P (PtdIns-4-phosphate), PtdIns5P (PtdIns-5-phosphate), PtdIns(3,5)P2 (PtdIns-3,5-biphosphate), PtdIns(4,5)P2 (PtdIns-4,5-biphosphate) and PtdIns (3,4) (PtdIns-3,4-biphosphate). PtdIns (4,5)P_2_ is the most well studied PI, accumulates at apical membrane and has been shown to interact with actin binding proteins [64,66,67] and Rac-Rop GTPases that control/coordinate the actin cytoskeleton and membrane trafficking at the tips of polar growing plant cells [48,68,69]. Alteration of these GTPases results in significant morphological and functional impairment in polar expansion. Second, PtdIns(4,5)P_2_, controls Ca^2+^ membrane channels that regulate Ca^2+^ gradients at the tip that are important for tip-based expansion and growth oscillations. Third, PtdIns are involved in regulating polarized membrane trafficking, *i.e.*, membrane flow between the Golgi Apparatus and plasma membrane including targeting secretory vesicles to the plasma membrane [70,71,72].

### 2.5. ROP/Rac GTPases

Small GTPases (Rho family GTPases) are integral components for vesicle budding, transport and fusion at specific targets and for establishment of polarity in eukaryotes [68,69,73,74,75]. They often work with cytosol and membrane vesicle proteins including SNAREs (soluble *N*-ethylmaleimide-sensitive factor attachment protein receptors) in targeted secretion activities [46,75]. In plants, Rho GTPases are found at the PM of the tips of expanding pollen tubes and root hairs and act in a switch-like fashion that cycle between the inactive cytosolic GDP-bound state and active membrane-bound GTP bound state [68,76]. In pollen tubes, it is believed that they promote polymerization or stabilization of F-actin in the subapical fringe. They may also regulate cortical microtubules, and serve as key players in both exocytosis and endocytosis [47,77].

### 2.6. Ca^2+^ Dynamics

Ca^2+^ is critical to polar expansion dynamics on many fronts both directly and as a secondary messenger [78,79]. In pollen tubes, the Ca^2+^ gradient at the tip is closely associated with secretion, actin dynamics (via actin-binding proteins) and the actions of Ca^2+^-dependent protein kinases [42]. Tip-high Ca^2+^ gradients in these and other polar expanding cells are typically coupled to a localized extracellular influx of Ca^2+^ [80]. This high Ca^2+^ level causes F-actin to depolymerize, in turn, stimulating exocytosis. As the tube tip expands, stretch-activated Ca^2+^ channels also open up, altering Ca^2+^ levels that affect targeted secretion and expansion at the tip. This phenomenon is also associated with growth oscillations noted in many pollen tube types. Tip-based Ca^2+^ gradients and their activities have also been noted in the polar expansion of root hairs [49], rhizoids protonemata and desmids. The Ca^2+^-responsive secondary messenger, calmodulin, has also been identified and is important in signal cascades involving Ca^2+^ in pollen tube growth [81]. It is also important to note that Ca^2+^ plays a key role extracellularly as it is necessary for increasing the rigidity of cell wall HG gels after PME activity (see *3.1.A*; [82]).

### 2.7. Reactive Oxygen Species

Reactive oxygen species or ROS has recently been found to be important in polar expansion phenomena including root hairs and pollen tubes [47]. ROS produced by a membrane-based NADPH oxidase (Nox) activates Ca^2+^ permeable channels at the tip that increase Ca^2+^ influx, a key component of polar expansion including actin dynamics and crosslinking of pectins in the cell wall [83].

## 3. Polar Growth Mechanisms in Early Divergent Plants

In early divergent plants such as the CGA and bryophytes, polar expansion serves multiple and remarkably diverse functions. These include adhesion, gravitropism, vegetative proliferation, habitat sensing, phototropism and photo-absorption maximization. Presently, no polar growth mechanism of these plants has been analyzed with the same level of detail as that performed for pollen tubes or root hairs. Yet, the great array of polar expanding cell types and the experimental attributes of their simple morphological forms make the early divergent plants superb systems for elucidating the subcellular processes involved in polar expansion. Likewise, these investigations hold much promise for deciphering the evolutionary ramifications of different cell expansion processes during the invasion of land by plants 450 million years ago and subsequent proliferation into modern terrestrial ecosystems. The following organisms represent just some of the polar expanding cells of early divergent plants:

### 3.1. The Charophycean Green Algae (CGA; Streptophyta)

The CGA or the Streptophyta represent the group of extant green algae that are most closely related and ancestral to land plants [84]. Within the six recognized clades of the CGA, polar expansion phenomena have been described in taxa of the Zygnematales, Coleochaetales and Charales, *i.e.*, the most “advanced” or the later divergent clades of the CGA. In some instances such as desmid shape regeneration, rhizoid formation in *Spirogyra* or hair cell production in *Coleochaete*, distinct and sometimes spectacular polar expansion occurs to produce morphologies about whose functional roles will require much more investigation.

The Zygnematales is the assemblage of the CGA that are distinguished by a conjugation-based mode of sexual reproduction. Despite this unique characteristic, recent phylogenomic studies suggest that these algae may very well be the closest extant ancestors to land plants [85,86]. Polar expansion mechanisms are quite common in this group with the most widely recognized forms exhibited by desmids. A desmid cell consists of two equal halves or semicells, each exhibiting the same size and shape, once fully mature. When a desmid divides, a new, small daughter semicell that is devoid of its taxon-specific shape forms and is attached to a fully formed or mature parent semicell. During post-cytokinetic morphogenesis, the daughter semicell ultimately expands and manifests the shape and size of the parent semicell. This process often requires a multipolar growth mechanism. That is, expansion during semicell morphogenesis occurs on more than one front of the developing semicell. The desmid that exemplifies multipolar growth during morphogenesis and has been well-studied is the beautiful *Micrasterias.* Each semicell of a mature *Micrasterias* is dissected into several major lobes that often can be further dissected into smaller secondary lobes. The post-cytokinetic development of these lobes occurs during semicell expansion and requires a targeted membrane transport system. This includes a complex Golgi Apparatus-derived network of vesicles carrying various primary cell wall precursors to multiple specific expansion sites on the cell surface, *i.e.*, each site representing one of the growing tips that will yield a future lobe [87,88]. Pectin, specifically high-esterified HG, is secreted at these growing tips. Subsequent PME-catalyzed de-esterification of the HG and Ca^2+^-complexing follow and lead to a rigidification of the wall [89]. These events are similar to those occurring at the tip of angiosperm pollen tubes and support the general model of polar growth whereby expansion is focused at a “soft” zone(s) of the cell wall that is strong enough to resist internal turgor pressure but elastic enough to allow for expansion on a narrow front [90]. A similar mechanism of pectin-based expansion has also been reported in the desmid, *Netrium digitus* [91].

Cellulose synthesis also plays a role in defining wall microarchitecture in the desmid multipolar expansion process. CesA activity has been demonstrated at both the indentations and sides of the expanding lobes [92]. These zones are removed from the HG secretion site at the lobe tips and are comparable to the subapical zone of a pollen tube where callose and cellulose are deposited. CesA was also identified in the cisternal peripheries of the Golgi bodies of *Micrasterias* and is apparently transported to the cell surface via Golgi-derived vesicles. A recent analysis of the *Micrasterias* transcriptome has also identified 30 genes involved in production of wall components including expansin and AGP-like macromolecules [93]. Overexpression of an expansin-like protein in transformed *Micrasterias* resulted in a loss of polarity during semicell morphogenesis.

The transport of the wall precursor-containing vesicles in *Micrasterias* is part of an actin-based cytoplasmic streaming network found in the peripheral cytoplasm [94]. Vesicle fusion sites at the plasma membrane are defined by regions of localized high concentrations of Ca^2+^ [95]. Experiments utilizing vibrating probe technology have shown that Ca^2+^ as well as other ion-channels may be necessary for polar expansion [96]. Two members of the Rab GTPase family and two members of the SNARE cycle have also been shown to be part of exocytosis. The Rab GTPases have been implicated in vesicle trafficking during morphogenesis [97] and the SNARE components most likely are involved with targeted vesicle fusion.

*Penium margaritaceum* is another desmid whose mechanism of cell wall expansion during morphogenesis has been recently described. [98,99,100]. *Penium* is a simple cylindrical unicellular desmid that produces only a primary cell wall consisting of a prominent, outer Ca^2+^-HG-rich layer, an inner cellulosic layer and a middle layer where pectin interfaces with the cellulose. HG secretion and cell expansion may be conveniently monitored using immuncytochemistry with live cells. Prior to cell division, a bipolar expansion initiates at the cell center or isthmus. Here, high esterified HG is secreted in a narrow band. As the HG is subsequently displaced outward toward both poles by newly secreted HG, it is de-esterified by PME, complexes Ca^2+^ and forms the rigid, Ca^2+^-complexed, outer cell wall layer. Cytokinesis takes place after significant expansion and ultimately yields two daughter cells, each with an old parent semicell and a smaller daughter semicell. HG secretion and cell expansion then continue at the pole of each daughter semicell in a unipolar fashion. 

In several species of the filamentous Zygnematalean genus, *Spirogyra*, another type of polar expansion has been observed. When a filament is wounded, cells immediately adjacent to the wound site begin to form rhizoids (Figure 2) [101]. The rapid developmental events associated with post-wounding rhizoid production entail alteration of the mechanical properties of a specific locus of the cell wall, focused wall precursor secretion at this zone, actin-mediated transport networks for delivery of new wall components and growth patterns in response to environmental cues including substrate sensing. A rhizoid protuberance first emerges from the periphery of the cell. The protuberance expands rapidly by tip growth and may begin to branch, usually when coming into contact with a substrate. Multi-branched or “rosette” rhizoids develop upon contact with a hydrophobic surface whereas non-branched rhizoids form on hydrophilic surfaces [102]. Our laboratory has noted that AGP-like components are produced at the rhizoid tip and may be key in the adhesion process (Figure 2). The exact components of the rhizoid wall have yet not been fully elucidated but callose-like and galactose-rich materials are common in the wall of the expanding rhizoid [102,103]. The morphogenetic process leading to rhizoid development is controlled both by a network of actin filaments and high Ca^2+^ influx at the polar tips [54]. Microtubules may also play a role in rhizoid development but this has been incompletely resolved [104]. 

In the Coleochaetales and specifically the genera, *Coleochaete* and *Chaetosphaeridium*, distinct hair-like projections or setae that may attain lengths of several millimeters are produced from cells of the thallus [105,106,107]. Each thallus cell has the potential for yielding a seta. Each seta consists of a basal collar from which the walled, tubular hair emerges. A long thin cytoplasmic projection fills the tube. The basal cytoplasm of the seta contains a chloroplast, large amounts of Golgi bodies and a distinct “setasome” consisting of a dense granular core surrounded by numerous vesicles. Application of pharmacological agents that affect actin and microtubules does not alter seta formation. However, Ca^2+^ is necessary for the process of seta elongation. Presently, no information is available about the polymers of which the seta cell wall is composed including those located at the expanding tip.

Within the CGA, the most thoroughly studied polar expansion mechanisms are those associated with the rhizoids and protonemata of *Chara* and it sister genera in the Charales. Significant interest has been generated here because these filamentous phases are highly gravitropic, are relatively easy to manipulate for experimental and microscopy-based studies and are very adapatable for space biology experiments [52,108]. Additionally, blue light exposure [109] and auxin [110,111] affect their growth patterns making these systems attractive tools for basic physiological studies. Both protonemata and rhizoids arise from the same cell type yet ultimately differentiate into polar growing filaments that are negatively gravitropic and positively gravitropic respectively. *Chara* rhizoids, the primary focus of many of these studies, have been shown to expand at rates of 100–200 µm/h. Their cytoplasm contains statoliths composed of barium sulfate that are specifically positioned in the cell by an extensive actin network. Upon exposure to gravity, a remodeling of the actin network and localized loss of apical microtubules cause a repositioning of the statoliths that subsequently influences the direction of rhizoid tip expansion. Stratification of cellular compartments is also prominent in the expanding terminal cell of the rhizoid [112,113]. The apical tip of this cell is highlighted by the presence of a Spitzenkorper. This structure consists of a dense aggregate of endoplasmic reticulum (ER) surrounded by a network of secretory vesicles. The Spitzenkorper is believed to control spatially-coordinated exocytosis. Its ER network serves as a reservoir for Ca^2+^ and a regulator of the tip-based Ca^2+^-gradient that is necessary for vesicle secretion and the spatial activity of actin-binding proteins [114]. The Spitzenkorper is also believed to be integrally involved in localized actin dynamics as its center contains actin, spectrin, profilin and actin depolymerization factor (ADF). The actin cytoskeleton is essential for acropetal movement of vesicles carrying new wall and membrane components to the growing rhizoid tip. Actin also controls the distribution of Ca^2+^-channels in the plasma membrane at the growing tip. This helps to establish a tip-high gradient of cytoplasmic Ca^2+^ necessary for focused exocytosis. Profilin is also involved in regulating actin dynamics as it most likely functions in keeping monomeric actin levels high and suppressing spontaneous nucleation that leads to actin filament formation. Spectrin may serve as the link agent in the ER aggregates in the Spitzenkorper. Other proteins, not associated with the Spitzenkorper, have been identified at the rhizoid tip. These include fimbrin which may help maintain the fine meshwork of apical actin and plasma membrane-bound integrin that most likely is involved in gravity perception/transduction and subsequent tip expansion [115]. 

The cell wall and its expansion dynamics in intermodal cells of *Chara* have been well-characterized and includes a distinct pectin-Ca^2+^ “cycle” [116]. At present though, little information is available demonstrating if this mechanism occurs at rhizoid or protonematal tips. 

### 3.2. Physcomitrella Patens and Mosses

Polar growth in bryophytes has been studied primarily in the protonemata phase of the gametophyte of mosses [117], particularly that of the model taxon, *Physcomitrella patens*. There are two types or stages of protonemata: (a) the chloronemata which consists of highly vacuolated cells containing many plastids and cross walls parallel to the long axis of the cell; (b) the caulonemata which possesses a basal vacuole, fewer plastids and cross walls that are transverse to the long axis of the cell. The chloronemata develops first from spores or adventitiously from the leafy gametophyte (e.g., after wounding or fragmentation). The caulonemata arises from the chloronemata and may undergo morphogenesis to yield the leafy gametophyte under the proper environmental conditions. Both types of protonemata exhibit polar tip expansion mechanisms. The *Physcomitrella patens* protonemata can be easily maintained in culture and grows at rates of up to 50 µm/h. Furt *et al.* [118] analyzed transformed protonemata with fluorescent proteins attached to proteins specific to various organelles and noted that caulonemata have 1.2–2.7 times more Golgi bodies than chloronemata but 1.3 to 6.2 times fewer plastids. Organelle gradients are notable in the tip of the apical expanding cell of the caulonemata. Here, a Golgi body-free zone and network of actin filaments occupy the first 1–2 µm of the cytoplasm of the growing tip. Mitochondria are not present in the first 2–3 µm and plastids and peroxisomes are absent from the first 9–15 µm. An actin network at the tip may be responsible for sorting out organelles from this zone. In the chloronemata, there is only slight organelle compartmentalization yet polar expansion still occurs. This indicates that compartmentalization of organelles may not be a universal requirement for polar growth.

In the protonematal apical cell, a dynamic equilibrium, *i.e.*, one that is constantly changing between monomeric and filamentous actin, is needed for polar expansion [119]. Recently, an array of proteins that is critical for actin dynamics has been characterized in *Physcomitrella*. Class XI myosins have been identified and function as the motors for vesicle delivery of wall material to the apex [55]. Profilin directly interacts with actin as well as poly-l-proline ligands found in the plasma membrane that are responsible for organizing actin [120]. These include class II formins that are localized at the apical tip and act to generate rapidly elongating arrays of actin filaments at the apex [55]. The Actin Interacting Protein1 (AIP1) and Actin Depolymerizing Factor (ADF) are also present with AIP1 enhancing actin polymerization and ADF regulating actin severing, both necessary for dynamic cycling of actin at the tip [121]. The Actin Related Protein complex, Arp2/3, and specifically the protein ArpC4, function to nucleate actin aggregates from monomers and initiate filament formation. It also has the capacity to translate directional cues like polarizing light for generating polar expansion [60]. BRICK1, a member of the Wave/SCAR complex activates the Arp2/3 complex and functions in the accumulation or stabilization of actin and other proteins required for polar growth [122]. While actin-based activity has been well studied in *Physcomitrella* [51], it may also be that microtubules also play a significant role in organelle movement. 

The cell walls of *Physcomitrella* and their protonemata contain cellulose, ß-(1-3)-glucans, HGs, RGI, mannan, xyloglucan, xylan and proteoglycans [123,124]. Lee *et al.* [23] also demonstrated the need for AGPs at the expanding tip of protonemata. A knockout of the AGP-core protein gene resulted in notably reduced growth. A potential role for AGPs in expansion includes acting as wall plasiticizers at the tip zone where wall remodeling is occurring. Alternatively, they may function in the localized unloading of Golgi vesicles or the deposition of wall polymers. 

### 3.3. Polar Expansion, Early Divergent Plants and Possible Roles

Tip growth has been described as a manifestation of a cell to invade a surrounding matrix in an efficient and flexible manner so as to respond to environmental cues [125]. With pollen tubes, root hairs and rhizoids, the material to be invaded is quite apparent (e.g., female reproductive tissue, soil and other substrate). However, for other products of polar expansion, functional roles are not so clear-cut. In *Spirogyra*, the production of rhizoids immediately after thallus wounding appears to be a rapid response mechanism for finding a suitable substrate on which to attach. *Spirogyra* species often bloom in large aggregate masses in ephemeral pools of standing or slowly running water. Maintaining close proximity to other thalli in the bloom would be advantageous for at least two reasons. First, the conjugation-based mode of sexual reproduction requires that the thalli of both mating types (*i.e.*, “male” and “female”) lie in close physical proximity to each other. Thalli of different mating types must make actual contact for gamete fusion to occur. Second, most taxa of the Zygnematles including many species of *Spirogyra* secrete gel-like polysaccharides outside of their cell walls. These polysaccharide-based gels hold the thalli in loose aggregates and represent a type of extracellular polymeric substance or EPS that is often associated with biofilm communities. EPS matrices are critical for (a) establishing communication between cells of the biofilm in order to support critical symbiotic associations and, (b) providing protective sheaths that guard against physical and biotic stresses. Rhizoid formation in *Spirogyra* may serve as a mechanism for assuring that thalli stay in close proximity in order that the collective EPS matrix may more effectively establish/maintain important communication networks with symbiotic microbes and accommodate better defense mechanisms against desiccation, pathogen attack and or herbivory. 

For desmids, the distinct, often multi-lobed, morphology provides a maximized surface area for light capture for photosynthesis. In virtually all desmids, the cell lobes formed by polar expansion are subsequently filled with lobes of the chloroplast(s) that significantly increase the surface area for light capture. This morphological trait is important in that most desmids live in shallow wetlands where light levels may be periodically low especially during periods of ecosystem disturbance and during seasons (e.g., summer) when competition for light with surrounding photosynthetic microbes and plants is keen. Maximizing surface area of a chloroplast becomes critical for light capture and photosynthesis. The mulipolar growth mechanism becomes the means for creating these lobes that provide the large light-capturing surface area. 

For the seta of *Coleochaete*, virtually nothing is known about their function or production. *Coleochaete* also grows in shallow wetlands or moist soils near wetlands. The polar-growing seta may be sensory for recognizing the direction of optimal light and the subsequent growth of the thallus toward that light (*i.e.*, a type of phototropism). It is also possible that the seta serve as flotation structures for thalli after they are dislodged from their substrate. 

The highly gravitropic rhizoids and protonemata of the Charales may have also evolved in response to habitat pressures. Many taxa of this CGA clade live in deep pools of freshwater where subsurface light levels drop off considerably. Many Charalean taxa are macroscopic, their thalli attain lengths of 0.25–0.50 meters (or even longer) and the thallus may initiate growth at considerable distances below the surface. With low or no light available at such depths, gravitropism-based probing of the habitat would represent a necessary sensing mechanism for determining the most likely direction for getting to the light. The simple filamentous components that grow by polar expansion would provide the exploratory vehicles. The protonemata that are negatively gravitropic would use the polar growth mechanism to grow up toward light and later undergo morphogenesis to yield the typical multicelled thallus. The rhizoids that are positively gravitropic would then serve as a means of anchoring the plant to the substrate. A comparable mechanism would most likely serve the protonemata of bryophytes. These simple filamentous structures would serve as an efficient mechanism for probing the terrestrial habitat before the commitment is made to produce the leafy gametophyte. Further insight into rhizoid evolution is available in a recent excellent review by Jones and Dolan [126].

## 4. Polar Expansion in Primitive Vascular Plants: Fern Rhizoids

Upon germination, fern spores yield rhizoids that also exhibit a polar expansion mechanism [127,128,129]. In *Dryopteris affinis*, rhizoids may grow at rates of 10–15 µm/h. As with many other of the aforementioned systems, polar expansion entails a notable compartmentalization of cytoplasmic constituents at the tip as well as a need for a critical level of Ca^2+^ at the rhizoid apex. There is a thickened 2–3 µm-long cytoplasmic cap at the tip that contains a large amount of secretory vesicles while vacuoles accumulate at the more basal regions. The apical zone also contains ROP-like proteins and annexin. Annexins may bind to plasma membrane phospholipids in a Ca^2+^-dependent manner and affect Ca^2+^ dynamics in this expansion zone. Endocytosis has also been described in rhizoids (129]). Finally, rhizoid expansion has been shown to be sensitive to nitrous oxide (NO) stimulation via cGMP [130]. 

## 5. The Pollen Tube

Like most polar growing cells, the pollen tube expands at a single narrow front (e.g., 5 µm wide in *Arabidopsis*) resulting in a tube-like product. This morphology enhances growth through the female reproductive tissue by restricting frictional forces to a small area. In pollen tubes, like in all walled plant cells, internal turgor pressure provides non-vectorial force driving cell expansion. This is controlled by the cell wall whose resistive properties are defined by its complex network of polymers, primarily polysaccharides and proteoglycans [2,131]. In a pollen tube, turgor driven expansion is resisted by the cell wall except at the growing tip or more specifically, at the sub-apical annular region situated immediately adjacent to the tube tip. Here, the cell wall consists of a less resistive, *i.e.*, “soft”, composite of polymers that, in turn, allows for regulated expansion in a polar or anisotropic manner (*i.e.*, tip growth). The presence of the soft wall at the tip and the expansion-resistant wall of the pollen tube shank are products of a highly compartmentalized and intricately regulated membrane trafficking mechanism [132]. The Golgi Apparatus consisting of many Golgi bodies processes and packages multiple wall precursors (e.g., pectins) that are transported to the growing tip. The wall precursors are secreted and the composite they form at the tip is resistant enough to prevent turgor-driven rupture of the protoplast but sufficiently elastic to allow for controlled expansion at the tip. The wall polymers are then modified via enzymatic activity (e.g., PME) that allows for restructuring of the polymeric composite. This, along with the manufacture of fibrillar wall polymers such as callose and cellulose, strengthens the cell wall and restricts expansion along the shank. Endocytosis also occurs during tube expansion and most likely functions in growth control and regulation of wall remodeling [33,47]. 

Membrane trafficking in the pollen tube is actin-mediated and is regulated by multiple actin binding proteins, calcium (Ca^2+^) and numerous cross-talking signal transduction mechanisms that include phospholipids, Rho-GTPases and Reactive Oxygen Species (ROS) [42,77]. The growth of pollen tubes in several Angiosperm taxa may exhibit oscillatory periodicities. The focal point of these expansion oscillations is at the transition zone of the hemispheric dome of the tip and cylindrical shank [133] and these growth phenomena are manifestations of mechanosensing feedback loops [4]. Multiple causes have been attributed to growth oscillations including changes in Ca^2+^ dynamics [3], exocytosis [134], pectin-Ca^2+^ dynamics [2,82] and NADP-oxidase/superoxide/peroxidase cross-linking of wall polymers [4]. For more detailed description of pollen tube growth, the reader is directed to multiple outstanding reviews [33,135,136]. Pollen tube growth in gymnosperms is also a polar expansion phenomenon. In comparison to angiosperms, the process is considerably slower, entails no callose cross wall formation and exhibits a different cytoplasmic streaming mechanism [137].

## 6. Root Hairs

Root hairs are unipolar cylindrical extensions of epidermal cells of roots. Root hair initiation requires localized alteration of wall microarchitecture and formation of a bulge. Like other polar expanding cells, there is a distinct compartmentalization of cytoplasmic constituents and reorganization of the microtubule and actin cytoskeleton. For more information, several excellent reviews are available detailing these systems [83,138,139,140].

**Figure 3:**
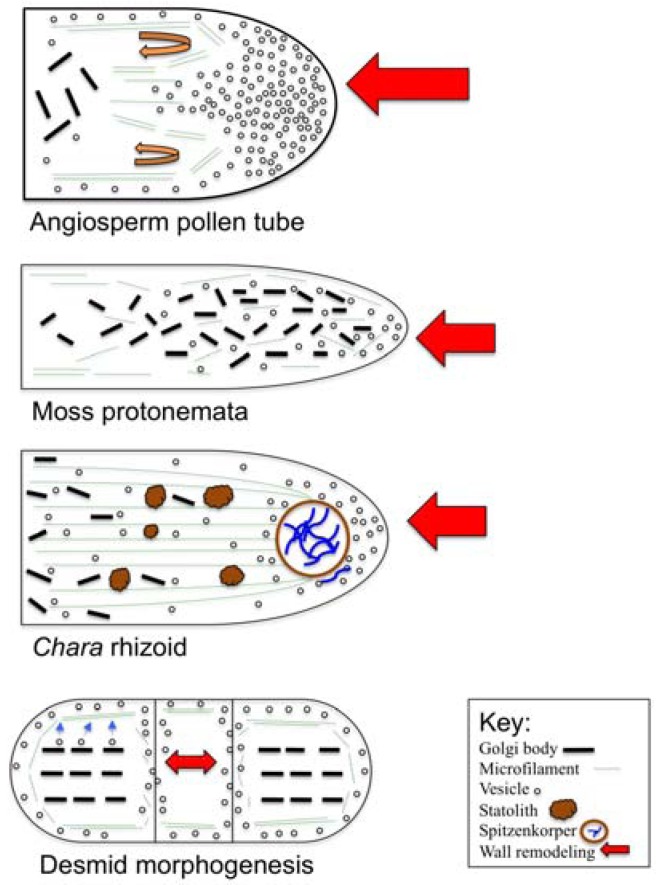
Comparison of the diverse polar and wall and cell expansion mechanisms in plants.

Figure 3 summarizes key examples of the polar wall and cell expansion mechanisms in plants. In angiosperm pollen tubes [132,133,134,135,136,137], Golgi-derived, wall precursor-containing vesicles travel to the tube tip along actin microfilaments in a reverse fountain-type mechanism (curled arrows). In the subapical zone of the tip, the wall precursors are released (*i.e.*, the site of wall remodeling). The Golgi bodies are positioned in a more distal region beyond the polar tip. In moss protonemata (e.g., caulonemata) [117,118,119,120,121,122], organelle zonation occurs but is more subtle with a larger proportion of Golgi bodies found near the tip. Actin microfilaments also transport vesicles to and within the tip zone. In *Chara* rhizoids [108,109,110,111,112,113,114,115], the growing tip contains an ER-vesicle aggregate known as the Spitzenkorper that serves as a site of actin organization and membrane trafficking. Also in the tip, statoliths, most likely made of barium sulfate, that are employed in gravitropism are held in place by the microfilaments. Vesicles are delivered to the tip from distal regions of the terminal rhizoid cell cytoplasm via the actin microfilament network. In desmids [87,88,91,92,93,94,95,96,97,98,99,100], polar expansion often occurs on multiple fronts. Large numbers of Golgi bodies are arranged in a symmetrical pattern in the cytoplasm and produce wall precursor-containing vesicles. The vesicles travel to the peripheral cytoplasm where they are transported around the cell in an actin microfilament-driven cytoplasmic streaming network. During polar expansion, the wall precursor-containing vesicles move to sites of polar expansion during pre- or post-cell division expansion.

## 7. Conclusions

Though polar expansion is far less common than “diffuse” expansion dynamics in plants cells, it is widely exhibited across the taxonomic spectrum of plants, yielding a diverse assortment of specialized cells with distinct and varied functions (Table 1). A comparison of general wall and cell expansion mechanisms is presented in Figure 3. It is apparent that some developmental processes are shared amongst most polar expanding plant systems. They include:

(1) Localized alterations (*i.e*., remodeling) to cell wall chemistry and structure are focal points for polar growth. These zones are produced via targeted cell wall secretion and/or alterations to pre-existing wall zones and results polymer composites that can resist internal turgor but allow for controlled expansion on a narrow front.
(2) Polar expansion requires new plasma membrane and cell wall material. This entails targeted secretion of endomembrane (*i.e.*, primarily Golgi-based) components that are transported/directed to the expansion tip via the actin cytoskeleton. This entails complex and integrated interactions of actin with actin-binding proteins that are in turn, regulated by various signal transduction molecules. The microtubule cytoskeleton may also play a significant role in the polar expansion process.
(3) Polar expansion directed by targeted secretion requires high levels and/or gradients of Ca^2+^.
(4) Many regulatory molecules and mechanisms are involved with polar growth. These include, phospholipids, Rho-GTPases and ROS, to name just a few or what is currently known.

**Table 1:** Examples of structures that utilize polar growth mechanisms in plants.

Structures	Taxonomic groups	Purpose	References
Pollen tubes	Angiosperms, Gymnosperms	Delivery of male gamete to female gametangium	[3,132,133,134,135,136,137]
Root hairs	Vascular plants	Absorption, anchoring, symbiosis	[138,139,140]
Protonemata	Bryophytes	Substrate “exploration”, anchoring	[117,118,119,120,121,122]
Rhizoids	Ferns, bryophytes, Charales	Absorption, anchoring	[108,109,110,111,112,113,114,115,127,128,129,130]
Hairs or setae	Coleochaetales	Unknown	[105,106,107]
Wound induced rhizoids	Spirogyra (Zygnematales)	Anchoring	[101,102,103,104]
Lobed cells	Desmids (Zygnematales)	Maximizing chloroplast surface area	[87,88,91,92,93,94,95,96,97,98,99,100]

In early divergent taxa like the CGA, polar expansion mechanisms are widespread and yield cells that perform diverse functions. The subcellular machinery needed for polar expansion of the ancestors of these algae that radiated onto and conquered land was subsequently adopted in a new set of roles in land plants. These include pollen tubes for sexual reproduction. Future research into the polar expansion mechanisms of early divergent plants should provide critical insight into the evolution of plant life over the past half billion years. 
